# Communities of practice for implementing methods to reduce involuntary care in intellectual disability care: Insights from tacit and experiential knowledge exchange

**DOI:** 10.3109/13668250.2023.2275225

**Published:** 2023-12-10

**Authors:** Esther H. Bisschops, J. Clasien de Schipper, Zina Salhi, Petri J.C.M. Embregts, Carlo Schuengel

**Affiliations:** a Department of Clinical Child and Family Studies, Vrije Universiteit Amsterdam, Amsterdam, Netherlands; b Tranzo, Tilburg School of Social and Behavioral Sciences, Tilburg University, Tilburg, Netherlands

**Keywords:** Implementation, tacit knowledge, intellectual disability, community of practice, involuntary care

## Abstract

**Background:**

Intellectual disability organisations in the Netherlands are seeking to improve clients’ quality of care by implementing methods that reduce involuntary care. This study described insights gained from sharing tacit and experiential implementation knowledge in Communities of Practice (CoP).

**Method:**

In a participatory research, managers, policymakers, experts-by-experience, support staff, and researchers participated in two CoP. Transcripts of the first meetings, focusing on tacit implementation knowledge and experiences in intellectual disability care, were analysed qualitatively.

**Results:**

Six themes and nine subthemes were found. One related to implementing methods in line with the Care and Coercion Act. Other themes were the quality of care dilemma, implementation determinants, organisational context, change in organisational culture, and implementation plans and strategies.

**Discussions:**

Findings reveal insights regarding the impact of implementing methods that reduce involuntary care on care professionals, management, and organisations. Through thinking together, CoP participants collectively learned about implementing methods in intellectual disability care.

In line with the Convention on the Rights of Persons with Disabilities (CRPD) (United Nations, 2006), care professionals and legislators across the world find that restrictive practices and involuntary care should only be used against serious risk of harm to self or others as a last resort, using the lightest possible form, and for the briefest possible time (Rickard et al., 2013). In the Netherlands, organisations changed their policies regarding the use and registration of restrictive measures and involuntary care (Frederiks et al., 2017; Schippers et al., 2018). In addition, legislation was changed, such as the introduction of the Care and Coercion Act (CCA) in 2020. This Act focuses on providing care on a voluntary basis to all people with intellectual disabilities. Involuntary care, ranging from severe to mildly restrictive, can only be applied in cases of serious risk or harm. The Act now applies to all clients receiving long-term care, regardless of whether they live in a care facility or at their own home (Care and Coercion Act, 2018).

These changes in policies and legislation led to the development of methods aiming to support care professionals in changing and improving care for clients with intellectual disabilities with respect to restrictive measures and involuntary care. Studies on the use of these methods in practice were promising on raising awareness and phasing out involuntary care (Bekkema et al., 2021; Embregts et al., 2019; Schippers, 2019). However, implementation appears to be hampered by a lack of understanding of how to implement new methods in care for people with intellectual disability successfully.

Long-term intellectual disability care is characterised by life-long care and support on multiple life domains for clients by professionals of various disciplines (Kersten et al., 2018). Implementation of new methods therefore affects a whole system of professionals, clients, and their relatives (Bisschops et al., 2022). For direct support staff, the pressures and demands of changing work routines, and habits can cause feelings of anxiety and uncertainty about the future (Nilsen et al., 2012; Wood et al., 2014). This may be particularly true when implementing methods aiming to reduce involuntary care, given that involuntary care is a response to perceived risks for their own and clients’ safety. Support staff reported that they use involuntary care to avert aggression, reduce imminent danger, or calm a client. They evaluated restrictive measures as necessary and even potentially helpful in fostering their clients’ development (Dörenberg et al., 2018). A lack of readiness for change might potentially complicate the implementation of methods that seek to reduce involuntary care.

To enhance the chances for successful implementation, Communities of Practice (CoP) may be considered. CoP facilitates exchange of knowledge and experiences (Wenger, 1998; Wenger et al., 2002). Participants who are tasked with developing implementation plans may engage with other implementers, deepen their knowledge, and learn from each other. This process could accelerate future implementation of innovations and stimulate quality improvement. A systematic review by Barbour et al. (2018) concluded that CoP are found to improve competencies of participants, enhance problem-solving and reflective practice, and support changes to organisations, programs, and policies. Although studies on the effectiveness of CoP are still limited, and to our knowledge, no study investigated the proliferation of CoP in long-term intellectual disability care just yet, CoP have been promoted as a useful strategy in sharing, promoting, and implementing evidence-based practices in general healthcare (Ranmuthugala et al., 2011).

A CoP is believed to be a strategy in which tacit and experiential knowledge and perspectives of professionals working in healthcare are being exchanged and combined with explicit knowledge and scientific evidence (Kothari et al., 2011). According to Polanyi (1966), tacit knowledge refers to individual knowledge, deeply rooted in action, commitment, and involvement in a specific context (Nonaka, 1994; Polanyi, 1966). Tacit and experiential knowledge are usually exchanged through informal channels and personal interactions, which require personal contact and trust (Kothari et al., 2011; McDermott, 2000). A CoP might be a way to create a safe environment for such learning, experimenting, and working towards a shared goal (Wenger et al., 2002). According to Wenger (1998), participants have their own individual, often incomplete, memories of previous efforts, and their unique way of bringing in their version of these efforts. Through participation in the CoP, participants are mutually engaged in a collective learning process in which the trans-personal knowledge concept of *thinking together* is at the core (Pyrko et al., 2017). In this concept of “thinking together” tacit knowledge is not only exchanged, but also (re)developed as people discover each other’s perspectives and experiences. New experiences are gained and added rather than being acquired and duplicated (Pyrko et al., 2017). It is still an empirical question to what extent CoP facilitate the exchange of tacit and experiential implementation knowledge, and in what way “thinking together” contributes to, first, understanding the issues affecting implementation within intellectual disability care, and, second, planning future implementation.

## Aim and research question

The aim of this study was to gain insight into implementation in intellectual disability care by examining the exchange of tacit and experiential knowledge in two CoP, which were set up to facilitate implementation of methods aiming to reduce involuntary care. The research question was: what were insights created through sharing tacit knowledge and experiences in CoP about implementation in intellectual disability care?

## Methods

### Context of the study

This study was part of a project to implement three methods that are substantively in line with the CCA in various organisations. The method “With other eyes” aims to enhance awareness among care professionals around the application of (in)voluntary care by observation and reflection, and invites to seek alternatives (Bekkema et al., 2021). The e-tool “Needs Assessment Framework” offers care professionals an easy-to-use online tool with video examples to help them reflect on clients’ perspectives in the use of involuntary care, while also helping them to enhance self-determination of people with intellectual disabilities (Embregts et al., 2019). The method MDET (Multi-Disciplinary Expertise Team) is a multi-component program aimed to reduce involuntary care with the use of multi-disciplinary consultation (Bisschops et al., 2022; Schippers, 2019).

To promote and study the implementation of these methods, researchers initiated two CoP (Wenger et al., 2002) with the aim of sharing knowledge, creating awareness, and insights about implementation, and working together in a planned manner. CoP-1 started in June 2019 to prepare participating organisations for the implementation of the methods MDET and “With other eyes,” and was still running during the data analysis of the current study. Due to COVID-19 in 2020 the focus of this CoP changed to implementing MDET only. CoP-2 started in October 2021 to design implementation strategies for the E-tool “Needs Assessment Framework” and finished in 2022.

### Participants

In CoP-1, six care organisations participated, in CoP-2 four organisations. Two of these organisations participated in both CoP. The care organisations varied in size from very large (14,000 clients living in areas across the Netherlands) to small (300 clients living in one area). They all provided long-term residential care for clients with severe to mild intellectual disabilities with challenging behaviour.

Care organisations chose employees who would be involved in implementing the three methods as CoP participants. In the first meeting of CoP-1, participants of organisations were policymakers (3) with experience in change processes or affinity with legislation concerning restrictive measures, frontline managers (3), behavioural consultants (two persons with a background in psychology and pedagogical science), and one support staff worker with experience in the method “With other eyes.” Also, three experts-by-experience from one organisation participated. They were persons with a mild intellectual disability who lived in care homes and worked as experts-by-experience who have been trained for this job. In this first meeting, five researchers were present. All but one policymaker and one manager were present in the second meeting. Of the four researchers in the second meeting, three also had been present in the first. In both meetings, students were present to observe. They did not contribute to our data collection.

In the first meeting of CoP-2, participants of organisations were policymakers (4) with an affinity for implementing the CCA, support staff workers who were assigned with the role of implementer in their care team (2), researchers (3), and one behavioural consultant. Experts-by-experience did not participate in this CoP. One of the researchers was only present to observe, as were two students.

The study was approved by the Ethics Committee of the faculty of Behavioural and Movement Sciences, Vrije Universiteit Amsterdam VCWE-2019-090R1. All participants received detailed written information about the study, including the possibility to leave the study at any time. They also signed a consent form before taking part in the study.

### Design and data collection

A Participatory Health Research design (ICPHR, 2013) was conducted within the CoP. The first and second meetings of CoP-1 were onsite, the meeting of CoP-2 was online due to COVID-19. In the first meetings of CoP-1 researchers organised discussions with the aim of retrieving tacit knowledge, previous experiences, and perceptions regarding implementation processes. Participants were invited to provide examples of successful and less successful implementation. In CoP-2 only the first meeting had this focus. Other meetings focused on discussing implementation determinants and strategies. Therefore, for the current study, data collected in the first and second meetings of CoP-1 and the first meeting of CoP-2 were analysed. Table 1 gives an overview of participants and topics.

**Table 1.** Overview of participants and topics.

*Community of Practice 1*		
Meeting 1	Care professionals (*n = *9) of various care organisations (*N = *6)	In three subgroups participants discussed prior implementation experiences (meeting 1C, 1S, 1B).
June 2019 (Onsite)	Experts-by-experience (*n = *3)	
	Researchers (*n = *5)^a^	
Meeting 2	Care professionals (*n =* 7) of various care organisations (*N = *6)	Participants of three organizations presented prior experiences with implementation and answered questions (meeting 2P).
September 2019 (Onsite)	Experts-by-experience (*n = *3)	Information about implementation interventions was shared with participants. Participants explored prior experiences with implementation interventions and discussed their experiences (meeting 2D).
	Researchers (*n = *4)^a^	
*Community of Practice 2*		
Meeting 1	Care professionals (*n =* 7) of various care organisations (*N = *4)	Participants discussed prior implementation experiences (meeting 1W).
October 2021 (Online)	Researchers (*n = *3)^a^	

^a^ Students (*n = *1–3) were also present to observe.

### Data analysis

All meetings were audio recorded and transcribed verbatim. Transcripts were uploaded in ATLAS.ti Windows (Version 22.0.6.0). A thematic analysis was conducted (Braun & Clarke, 2006). The first author re-read the transcripts several times before starting inductive coding. Significant lines were coded descriptively, resulting in 116 initial codes and seven code groups. The code groups were discussed with the second author to gain insight in the underlying story, which could represent significant themes concerning implementation.

Hereafter, the third author read the transcripts and reviewed codes and code groups, which led to suggestions for adding, merging, and deleting codes and code groups. After discussing these changes, the first and third authors reviewed the transcripts with the new codes for a second and third time until consensus was reached on 69 codes and 13 code groups. The code groups were then renamed and reorganised to six themes and nine subthemes (Results, Table 2). Overall conclusions and implications were discussed by the first author with the other authors. Quotes were originally in Dutch. The first author translated these to English, and checked these translations at Deepl.com.

**Table 2.** Overview of themes and subthemes in the CoP exchanges.

Themes	Subthemes
Implementing methods in line with the CCA	-
Quality of care dilemma	-
Implementation determinants	Hindering and facilitating factors
	Stakeholder engagement
	Clarity on implementation goals
	Motivated and engaged professionals
Organisational context	Impact of organisational context
	Top-down implementation
	Bottom-up implementation
Change in organisational culture	Culture change
	Coaching implementation and change
Implementation plans and strategies	-

### Positionality and reflexivity

The first author has been working as behavioural consultant (in Dutch: orthopedagoog) in care for people with intellectual disabilities for 20 years. She is specialised in treatment of clients with challenging behaviour who faced multiple forms of restrictive measures. She was involved in the implementation of a Dutch treatment model (Triple-C; Tournier et al., 2020) including training activities, and advising support staff and colleagues. These experiences allowed her to easily recognise the topics discussed in the CoP, which was an advantage to quickly empathise with the participants’ experiences. To counter bias and maintain objectivity, meetings of the CoP were prepared for and attended by at least one other researcher with limited experience in implementation in care for people with intellectual disabilities. In addition, data were analysed with the third author, who had no experience with implementation. Also, the results were discussed with the second author who attended the meetings of CoP-1.

## Results

Table 2 gives an overview of the themes and subthemes found.

### Implementing methods in line with the CCA

The introduction of the CCA prompted care organisations to implement new methods aiming to reduce involuntary care. Participants identified a difficult situation for support staff. Due to the new legislation support staff might become more aware of their use of involuntary care in clients’ care and support. Since this is no longer legitimate, and methods in line with the new Act need to be implemented, support staff workers might fear for their own safety, because new ways of working might give clients with challenging behaviour too much freedom. Participants suggested that this should be discussed with direct support staff before implementing methods that aim to reduce involuntary care.

Because it also brings fear: If we are no longer allowed to suspend someone who has just threatened me with a knife, where do I stand? So you have staff who find it scary. And you can't say we're not talking about that, because this is now the new law. (Policymaker, meeting 1S)

### Quality of care dilemma

According to CoP participants, support staff did not have much time to spare beyond their care duties. However, they also felt responsible to provide clients with optimal care and felt compelled to implement innovations that would enable them to do so better. This resulted in support staff being faced with a so-called “quality of care dilemma,” meaning that adequately meeting clients’ short-term needs, by working their shifts to provide daily care and support in clients’ homes, competes with serving clients’ long-term needs, by attending meetings and spending time outside the clients’ homes, in information meetings and trainings.

The moment you are very busy and work a lot of shifts, the willingness to immerse yourself in other things diminishes. You are less inclined to go to such an information evening in your free time. (Support staff worker, meeting 1C)

Reducing time constraints was therefore seen as paramount to reduce this dilemma. Insights were that support staff would have ideas on how to organise this, in addition to frontline managers who were working within the constraints set by higher management. Nevertheless, if managers freed up direct support staff from working with clients, they could still feel guilty as clients would be affected by facing substitute workers and unknown support staff.

A lot of essential time for the client is lost. And I think that's something you often have to deal with on the work floor. (Support staff worker, meeting 1C)

When implementing innovations in intellectual disability care organisations, frontline managers should need to take into account the quality of care dilemma support staff have to cope with.

Knowing what it does to colleagues on the floor. Who are already busy with all kinds of things and are actually tiptoeing around a staff shortage and yet another person dropping out because he or she has been struggling for too long. There is just so much more going on that, for a lot of colleagues, this is really something like “oh dear, that's going to come as well.” I think we have to make sure that we can put the interests of the client first with this new implementation. (Support staff worker, meeting 1C)

### Implementation determinants

#### Hindering and facilitating factors

Hindering and facilitating factors can be recognised in many of the themes and subthemes. Examples were the overburdening and stress of care professionals as well as the frequent crises due to challenging client behaviours. In these situations, support staff showed resistance to implementing innovations. Also, a lack of information and communication were mentioned as barriers. Examples of facilitating factors were the organisation’s commitment to an innovation, embedding the innovation in regular work processes, investigating the resistance of support staff teams, and providing them the attention and resources they need to innovate.

#### Stakeholder engagement

Engagement of stakeholders (support staff, clients, or their representatives) was believed to facilitate implementation. According to the CoP participants, engaging support staff to determine which method will be implemented, giving them time to discuss their doubts, resistance, ideas and find their own solutions, promoted the likelihood of successful implementation. An expert-by-experience added that openness about implementing innovations to clients with intellectual disabilities would potentially improve this even more.

I think there should be much more dialogue with clients and support staff. Like, this is going to change: what does that mean. What does that mean for support staff. And also openness towards clients. That openness gives me the feeling of “I matter,” which also makes it much easier for me to understand. (Expert-by-experience, meeting 1S)

However, involving clients and their representatives took quite some time and effort. Due to their cognitive limitations, clients with intellectual disabilities were only partly able to participate and decide for themselves. However, attempts to reach, engage, and inform clients’ representatives often failed. If relatives and representatives participated, ideas about good care were often specific from one client to another. Also, family members of the same client not always agreed on what was important for their relative. As a result, engaging clients and their representatives in implementation took considerable effort, reducing support staff’s motivation to use innovations.

#### Clarity on implementation goals

Being aware of the need to improve care by implementing innovations, knowing the aim of innovations, and making a conscious choice for a particular innovation were discussed as conditions that facilitate implementation.

I think it's important in an implementation that the people who are on the work floor can consult each other. Is this the method from which we all say, yes this is good for all our clients and we can work well with this in the long run. (Support staff worker, meeting 2d)

Participants’ experiences, however, were that the reasons for implementing a particular innovation were not clear. Implementation processes were ad hoc, following a hype of a new popular method, and lacking careful consideration and coordination. Implementing the innovation often became “a checkmark to be set by a certain date” and missed the point of actually improving clients’ care. The danger here was that support staff became “implementation fatigued” as they were constantly confronted with innovations they do not see any benefit in.

P: “People get change-tired, and then they no longer take it seriously” R: “They are getting a little negative about possible future implementation?” P: “Yes.” (Policymaker and researcher, meeting 2p)

Knowing implementation goals beforehand was considered helpful. For example, goals such as employee satisfaction, improvement in quality of care, or normalising a method in daily routines. However, goals were often not sufficiently clear for proper evaluation, and participants wondered how these goals could be determined.

#### Motivated and engaged professionals

Motivated and engaged care professionals who are enthusiastic about the method could be of great value in the role of “implementation champion” and help moving implementation forward. Participants mentioned three conditions for making the use of motivated, engaged professionals as champions in implementation successful: first, these care professionals needed to have qualities and competences to convey their enthusiasm well. They also needed to be sufficiently facilitated by their managers and given the permission to act with a clear assignment. And finally, expectations and the reason why they were approached for this role needed to be clear for these champions. In discussing why this was important participants pointed out that using champions to facilitate implementation could also backfire on the whole team. Heralding champions may unwittingly fuel a competitive team climate, which would be counterproductive for achieving collective action.

That's the champion, who always does exactly what that manager asks. Well, I have experienced this first-hand and it was really very counterproductive. And she was also completely excluded from the team. So everything she proposed was a foregone conclusion: we're not going to do that. The champion became really detached from the team. (Policymaker, meeting 2D)

Participants agreed that champions should not only be enthusiastic about the innovation, but also have connecting qualities to work with a team during an implementation process.

We looked for someone who was actually very connected to the team, regardless of whether he or she carried a vision or not. That person is connected, has contact with everyone. In my experience, that was very valuable. (Policymaker, meeting 2D)

### Organisational context

#### Impact of organisational context

If a care organisation did not have a clear vision, or did not clearly articulate why an innovation should be implemented organisation-wide, teams tended to follow their own path. Broad support of the Board of Directors for a course of action shared with care professionals and managers helped implementation.

We do not have a clear vision of what we are going to do with it. Plus, there is still a lack of broad support. It is started very enthusiastically somewhere, but it does not yet touch the entire organisation. In that case, it will not be properly secured. (Support staff worker, meeting 1W)

Organisational issues could also undermine implementation at the scale of individual teams. Care teams differ in their developmental phases, as some teams have more experienced support staff than others.

Then it is rolled out as if every team is at the same level in terms of development. No thought is given to that. I would like it to be customised. That maybe there is an organisation-wide plan, that is needed, but that you do start looking at how can we then translate that on a tailor-made basis. (Policymaker, meeting 1S)

#### Top-down implementation

In the case of top-down implementation, the decision to implement an innovation was taken by the board or management of the organisation. They set a framework to improve quality of care, which gave clarity to managers and care professionals about organisational goals. However, participants mentioned that the Board did not always know what is feasible in implementing innovations.

It is a large organisation and new methods and innovations often come from above. I'm on the work floor, as a care provider there's not much you can do or say about that, you know. You get something imposed on you every time and then things come up that aren't going well, (…) I think, let's have people from the work floor start working on it in the initial phase, to do such an implementation or such an innovation, because they know what is feasible in practice and what is not. (Support staff worker, meeting 1C)

Another issue that came up as disadvantage in top-down implementation was the gap between guidelines, policies, and innovations developed by policymakers, especially in large organisations, and their use in practice.

Over the past few years, I've come across a lot of people who like to develop things and then, once it's developed, think: so, we've done that nicely. And then it ends up in the cupboard. People just don't like translating and securing it within the processes. Developing something is really the start. But implementation often takes much longer. (Manager, meeting 2p)

Finally, top-down implementation might generate resistance when support staff feel overwhelmed or experience a lack in facilitation. For example, the Board of one organisation decided to interview each individual client before filling in a questionnaire in their support plan, which is in line with the CRPD (United Nations, 2006) goal of enhancing clients’ self-determination. Support staff were given a deadline by which this must be completed. This led to chaos and stress because it was not feasible to properly interview each client.

Everything had to be done very quickly and all those clients had to be rounded up and conversations had to be planned with them, which didn't work at all. Which eventually led to a situation where the goal was lost. So the goal became more about filling in the lists on time than about giving clients more control over their own lives. (Behavioural consultant, meeting 2p)

#### Bottom-up implementation

Bottom-up implementation often started with a small enthusiastic group of care professionals who wanted to act quickly. This was found advantageous because implementation started where the innovation was meant to be used: in the context of support staff teams and group homes. Moreover, this gave the opportunity to try out innovations on a small scale before introducing it widely in the organisation.

What we often do within [organisation] is just go and do it, with a particular team, or group. See if it becomes a success. What works, what doesn't. And if a lot has to be arranged for it, talk about that positive experience with a management team, or other people who have to decide or allocate resources to it. But we often start by doing. (Manager, meeting 1B)

Participants admitted that in these bottom-up processes well thought-out implementation plans were not present yet. Some participants assumed that if the method being implemented worked on a small scale, and was well received, implementation plans could be developed afterwards to scale up and secure it within the organisation’s structure. Other participants had the experience that this did not work out well, particularly when the managers and Board of the organisation were not facilitating this.

What we are good at is getting new things started. And full of enthusiasm about a particular topic actually go for it. But what's tricky is being able to keep the focus. That's the most complicated thing: how can this be secured long-term? (Behavioural consultant, meeting 1W)

Participants had the experience that bottom-up implementation processes worked best in small organisations or on a small scale. Best practices could lead to snowball effects because successes were easily communicated throughout small organisations.

Another disadvantage that CoP participants mentioned with bottom-up implementation was the “implementation gap” between a small group of care professionals with specific knowledge about an innovation moving forward very quickly and the majority of care professionals who could not catch on because they did not have enough information, or did not know until late that an innovation was being implemented.

Someone has immersed himself in it so deeply that he knows all the ins and outs, so then the implementation goes so fast that the rest can't keep up with that pace. (Support staff worker, meeting 1W)

### Change in organisational culture

#### Culture change

CoP participants experienced too little collaboration within intellectual disability care organisations to implement improvements for direct support staff. They indicated successful implementation needs a change in the culture of care organisations. Organisations should not only implement innovations on the work floor, but also need to empower frontline management to educate themselves on “change processes and how to manage them.” This should enable them to take the contextual determinants in implementation processes into account and provide tailored solutions to support staff teams.

It's also a kind of cultural shift you have to make. (…) I would find it desirable if those managers would get a bit more uh, knowledge about what is that, change management, and what does that do to people when they have to do another change. (Policymaker, meeting 1S)

#### Coaching implementation and change

Participants suggested co-learning of managers and support staff, as this could lead to a team-development plan, which could include implementing innovations. However, the experience was that without coordination from someone outside the team, co-learning and developing such a plan was not successful. Teams might need a team coach specialised in change management or implementation, because these processes need mutual trust among managers and support staff to be able to say anything without further consequences.

#### Implementation plans and strategies

CoP participants agreed that an implementation plan, especially with tailored implementation strategies to fit the needs of the particular context, would be facilitating. However, many experienced that there was no implementation plan, or the content of this plan was unknown, which was a barrier. In the rare cases in which an implementation plan was present, the plan was often not clear, or not shared within the organisation, leaving support staff overwhelmed with implementation strategies that did not fit their work schedule or needs.

CoP participants discussed ideas on how this could be improved in the future. One suggestion was that support staff should ask for the implementation plan themselves when managers instruct them to implement an innovation.

Perhaps it also indicates that staff too are insufficiently aware that they need to hit the brakes, and ask for the plan. They just get going with all their good intentions. And so you can lose a lot with that actually. (Researcher, meeting 2p)

Another idea was to use or develop various implementation interventions so that customisation can be provided, tailored to support staff teams, or target groups you want to reach. One participant suggested using a “toolbox”:

As for support material, basically you should always just have a toolbox that people can draw from. But what they use from it, that depends a lot on where the team stands (Behavioural Consultant, meeting 1B).

Regarding future implementation of methods that reduce involuntary care, participants suggested clients, relatives, and support staff should be involved in designing an implementation plan. Starting early and knowing at the start how the new method will be secured in the organisation was found important. The plan should also be clear about which people have a key position, and which stakeholders should be involved. Besides designing tailor-made implementation interventions, there should be a strategy and timeline. The power of repetition was mentioned to remind support staff that a new way of working is required. Finally, participants mentioned that technical systems should work and that specialists need to be available who can prepare these systems.

## Discussion

This study aimed to understand issues affecting implementation in intellectual disability care, especially when implementing methods to reduce involuntary care. In two CoP, tacit knowledge and prior experiences with implementation in intellectual disability care were shared by CoP participants, who were a diverse group of managers, policy staff, experts-by-experience, direct support staff, and researchers. The findings reveal valuable exchanges and detailed insight of the issues at hand in long-term intellectual disability care during implementation, such as difficulties with implementing methods in line with the CCA, a quality of care dilemma, and hindering and facilitating factors. Also, ideas to improve implementation were discussed, such as support for organisational change and planning for implementation, which may help care organisation to prepare for future implementation projects.

First, it became clear that participants usually were not aware of the existence or content of an implementation plan. Also, making an implementation plan was postponed as the organisation first wanted to test on a small scale whether the innovation would work well. Previous research suggested that making such a plan with clear goals, together with care professionals, and approved by the management or Board, could be relevant (Voss et al., 2021). Based on their experiences, the CoP participants expected that a plan with tailored implementation strategies would be beneficial. This insight aligns with published examples of successful implementation based on such plans (e.g., van Schijndel-Speet et al., 2013, 2014) and efforts to provide online tools to choose and tailor implementation strategies, such as the CFIR-ERIC tool and the ItFits toolkit (Powell et al., 2015; Vis et al., 2023; Waltz et al., 2019).

Second, working with motivated engaged professionals, also referred to as “implementation champions’, or people who use their enthusiasm to bring innovations further was discussed in the CoP as a potential useful strategy. This is also evident in a study by Kaasalainen et al. (2015) in which nurse practitioners were very successful as change champions in implementing pain protocols in long-term elderly care. However, the participants in the CoP experienced this strategy could also backfire if the use of these champions was not sufficiently facilitated and explained within a team or organisation. This is in line with the debate Albers et al. (2020) started about the role of implementation support practitioners (ISP) as a way to effectively build the implementation capacities across healthcare organisations. Although knowledge and attitudes required for the role of ISP were presented in a systematic review (Bührmann et al., 2022), little is known about relevant knowledge, skills, competences, and attitudes required for implementation in intellectual disability care. Also, little is known about the contextual factors needed for ISP to do their work in a way that adds value to individuals and organisations engaged in implementing innovations (Albers et al., 2020). This was reflected in the dialogue in the CoP in which participants discussed that not only enthusiasm and knowledge about the innovation is important in implementation processes, but also knowledge about mechanisms of change, and being able to lead teams, clients, or their representatives and other stakeholders in the process of this change. Whether implementation champions recruited from local teams or implementation support practitioners coming from outside those teams are more effective may therefore be an important empirical question.

Third, the pros and cons of bottom-up and top-down implementation directions led to considerable debate. Although bottom-up processes had the advantage of innovations being tried out directly in daily practice, disadvantages with regard to scaling up the innovations were mentioned. Especially in large organisations, communication about successful innovations and the knowledge gap between early adopters and the vast late majority (Rogers, 2002) was discussed as barriers to wide implementation. Well thought-out implementation plans and strategies, such as training, were suggested to prevent communicative problems, which is also underscored in a systematic review (McArthur et al., 2021).

In top-down implementation, CoP participants mentioned that having a clear vision or framework to work with helped teams stay focused on the goals set by their organisation. A barrier in top-down implementation was the gap between policies, methods, innovations, and their practical use. Also “change fatigue,” because of being confronted with too many innovations to implement, was mentioned as a barrier. This was found in previous research as well (Berta et al., 2010). The quality of care dilemma that came up in the CoP could also be a barrier in top-down implementation processes. When organisations unveil innovation plans, the dilemma of spending time on improving quality of care in the long run, versus spending time to provide immediate quality of care to clients by working shifts, can be overwhelming for care professionals. A similar problem was pointed out in community nursing services in the United Kingdom (UK), where frontline nurses often felt frustrated that they were unable to implement high-quality care in accordance with policies without the appropriate resources (Haycock-Stuart & Kean, 2013). According to Bentur and Sternberg (2019), the synergy of top-down and bottom-up implementation strategies might contribute more than either strategy alone in addressing challenges in complex care organisations.

Fourth, because care professionals and clients with an intellectual disability are directly affected by new methods and change in work routines, engaging them in the choice of methods to be implemented and in designing implementation strategies was mentioned in the CoP as facilitating factor. People with intellectual disabilities, such as the experts-by-experience involved in CoP-1, want to give their opinion concerning choices, application, and evaluation of innovations (Wolkorte et al., 2019). Involving them in choices, and increasing self-determination and autonomy (Ryan & Deci, 2000), is in line with the principles behind the CCA in the Netherlands. It is therefore of great importance to involve clients’ perspectives in implementation of methods that seek to reduce involuntary care and promote care on a voluntary basis (Bisschops et al., 2022).

### Collective learning processes in the CoP

Based on the results of this study, we reflected on how the exchange of tacit and experiential knowledge in a CoP contributed collective learning (Pyrko et al., 2017). First, in discussing what implementing methods to reduce involuntary care could mean for support staff, participants of the CoP became collectively aware of difficulties and dilemmas these care professionals face when new methods are being implemented. The importance of discussing this in organisations and with teams became clear. Becoming aware of difficulties and dilemmas is essential for the development of critical thinking, and to make decisions based on professional values (Eyal, 2018, July 2-4), which will help in carefully balancing out the investments to be made while implementing methods to reduce involuntary care.

Second, in discussing barriers, facilitators, advantages, and disadvantages in implementation the concept of “thinking together” in the CoP led to supplementing each other's tacit knowledge and experiences. For example, adding to each other’s knowledge in discussing implementation strategies such as choosing consciously for a particular method, stakeholder engagement, and empowering management. When preparing for an implementation process, thinking together in a CoP may help in making determinant analyses, which are considered an important prerequisite for tailor-made implementation processes (Damschroder et al., 2009; Fleuren et al., 2004; Steenbergen et al., 2019).

Third, discussions in which CoP participants disagreed led towards in-depth conversations, in which collective learning processes were most prominent. By expressing points of view, interesting dialogues unfolded, which led to new perspectives and re-evaluation of beliefs and expectations. This is in line with the idea of McDermott (2000) that when care professionals discuss conflicting opinions, they reflect on the current situation and on their experience, view the topic “through the eyes” of different perspectives, and use those insights so that a new, creative idea can emerge (McDermott, 2000). For example, the discussion that arose after one participant claimed that the use of “implementation champions” was a valuable implementation strategy, while someone else experienced this as counterproductive. In the in-depth dialogue that ensued, it became clear what might be crucial conditions for the deployment of these champions. Without this contradiction, this tacit knowledge would probably not have surfaced.

Finally, the interactions in the CoP led to thought experiments in which new ideas came up that participants can benefit from when implementing new methods in their organisations. Such as, for example, writing an implementation plan in advance of each implementation, and the importance of sharing this plan with care professionals and other stakeholders, or the use of coaches who can guide implementation processes.

### Limitations

First, although this study touches upon many, in our view, important topics concerning implementation in intellectual disability care, it was not the aim of this study to provide a complete overview. Our focus was on exchanging and collectively learning from tacit implementation knowledge and previous experiences of care professionals and expert-by-experience. Therefore we assume not all relevant topics concerning implementation in general were discussed. For example, topics such as scaling up and sustaining innovations in normal routines of everyday work are known as important in implementation. Although participants did mention this briefly, they did not discuss this in detail, probably because they had little experience with it.

Second, CoP participants had no or very little experience with implementing methods that reduce involuntary care, probably because these methods hardly existed before or are not well-known. Specific determinants concerning implementing such methods may not have surfaced.

Third, the main purpose of this study was to gain insights in tacit knowledge and experiences, and not to address collective learning processes explicitly. This allowed us to only explore the data for this purpose, without being able to conduct an in-depth investigation of collective learning processes in the CoP.

## Conclusion

Two CoP were set up with the aim to prepare for implementing methods that reduce involuntary care. Exchanging tacit knowledge, and experiences with implementation in intellectual disability care, by managers, support and policy staff, experts-by-experience and researchers revealed implementation insights that may be useful in future implementation. Relevant themes were implementation of methods in line with the CCA, a quality of care dilemma, implementation determinants, organisational context, change in organisational culture, and implementation plans and strategies. These insights and knowledge are valuable for policymakers, managers, and implementation coaches setting out implementation pathways in intellectual disability care organisations.

Setting up a CoP in which participants share a common goal, and exchange tacit knowledge, perspectives, and prior experiences is considered a promising way to collectively learn about implementation in intellectual disability care. Employing such a method may be relevant in light of the limited evidence base for implementation strategies in this field, especially for high-stakes issues such as involuntary care.
